# Development and Characterization of a Sin Nombre Virus Transmission Model in *Peromyscus maniculatus*

**DOI:** 10.3390/v11020183

**Published:** 2019-02-21

**Authors:** Bryce M. Warner, Derek R. Stein, Bryan D. Griffin, Kevin Tierney, Anders Leung, Angela Sloan, Darwyn Kobasa, Guillaume Poliquin, Gary P. Kobinger, David Safronetz

**Affiliations:** 1 Department of Medical Microbiology and Infectious Diseases, University of Manitoba, Winnipeg, MB R3E 0J9, Canada; warnerb@myumanitoba.ca (B.M.W.); darwyn.kobasa@canada.ca (D.K.); gpkobinger@gmail.com (G.P.K.); 2 Zoonotic Diseases and Special Pathogens, National Microbiology Laboratory, Public Health Agency of Canada, Winnipeg, MB R3E3R2, Canada; derek.stein@canada.ca (D.R.S.); bryan.griffin@canada.ca (B.D.G.); kevin.tierney@canada.ca (K.T.); anders.leung@canada.ca (A.L.); angela.sloan@canada.ca (A.S.); guillaume.poliquin@canada.ca (G.P.); 3 Department of Pathology and Laboratory Medicine, University of Pennsylvania School of Medicine, Philadelphia, PA 19104, USA; 4 Centre de Recherche en Infectiologie, Centre Hospitalier Universitaire de Québec, Université Laval, Quebec City, QC G1V 0A6 Canada

**Keywords:** hantavirus, Sin Nombre virus, deer mice, *Peromyscus maniculatus*

## Abstract

In North America, Sin Nombre virus (SNV) is the main cause of hantavirus cardiopulmonary syndrome (HCPS), a severe respiratory disease with a fatality rate of 35–40%. SNV is a zoonotic pathogen carried by deer mice (*Peromyscus maniculatus*), and few studies have been performed examining its transmission in deer mouse populations. Studying SNV and other hantaviruses can be difficult due to the need to propagate the virus in vivo for subsequent experiments. We show that when compared with standard intramuscular infection, the intraperitoneal infection of deer mice can be as effective in producing SNV stocks with a high viral RNA copy number, and this method of infection provides a more reproducible infection model. Furthermore, the age and sex of the infected deer mice have little effect on viral replication and shedding. We also describe a reliable model of direct experimental SNV transmission. We examined the transmission of SNV between deer mice and found that direct contact between deer mice is the main driver of SNV transmission rather than exposure to contaminated excreta/secreta, which is thought to be the main driver of transmission of the virus to humans. Furthermore, increases in heat shock responses or testosterone levels in SNV-infected deer mice do not increase the replication, shedding, or rate of transmission. Here, we have demonstrated a model for the transmission of SNV between deer mice, the natural rodent reservoir for the virus. The use of this model will have important implications for further examining SNV transmission and in developing strategies for the prevention of SNV infection in deer mouse populations.

## 1. Introduction

Hantaviruses are a family of enveloped, negative sense RNA viruses with a tri-segmented genome of the order Bunyavirales. They are the causative agents of two distinct diseases in humans: hemorrhagic fever with renal syndrome (HFRS) caused by Old World hantaviruses found mainly in Europe and Asia and hantavirus cardiopulmonary syndrome (HCPS) caused by New World hantaviruses in the Americas [1]. While disease caused by hantaviruses was described several decades prior, the first isolation and characterization of a hantavirus occurred in the 1970s when Lee et al. isolated and described the virus responsible for Korean hemorrhagic fever, which they named Hantaan virus (HTNV) [2]. The first discovery of hantaviruses causing HCPS was in the early 1990s when Sin Nombre virus (SNV) was identified following an outbreak of HCPS in the southwestern United States [3].

In North America, SNV is the main causative agent of HCPS, responsible for greater than 600 infections in the USA and 120 infections in Canada with fatality rates of 35–40% [4,5]. HCPS is a severe disease characterized by fulminant respiratory failure and cardiogenic shock, and the clinical course begins following an incubation period with a mean of 14–17 days, ranging from 9 to 33 days [1]. The disease progresses through phases that can differ in severity depending on the patient and the pathogen responsible. Once symptoms develop, HCPS progresses rapidly, with most hospital admissions occurring 3–6 days following the onset of symptoms and most fatal outcomes occurring within 2 days of hospital admission [1]. While several vaccine formulations have been developed and tested for HFRS, there are no currently approved vaccines or treatments for HCPS [6].

Hantaviruses are zoonotic viruses mainly carried by rodents, bats, and insectivores that cause a persistent, asymptomatic infection in their reservoir hosts. SNV is carried by deer mice (*Peromyscus maniculatus*), and human infection occurs following accidental exposure to the virus found in contaminated animal excreta and/or secreta [7]. Studying SNV infection can be difficult due to the need to passage the virus in its rodent host to maintain virulence and infectivity [8]. The need for viruses to be taken directly from infected rodent hosts appears to be a common trend for at least two different hantaviruses, as cell culture adaptation of Puumala virus (PUUV) has been shown to result in mutations that alter its ability to infect its rodent host [9,10]. Similarly, passaging SNV in vitro results in mutations in the RNA-dependent RNA polymerase leading to a loss of virulence [8].

The transmission of SNV between deer mice has also not been well characterized, and previous studies have either shown very low transmission rates or have not been able to elucidate how transmission primarily occurs [11,12]. Additionally, the shedding of SNV is sporadic and intermittent, and little is known about what influences the temporal nature of SNV infection and the transmission of the virus [13]. It is thought that the transmission of SNV occurs primarily via one or more horizontal mechanisms and predominantly between male deer mice. While the transmission of SNV remains poorly understood, studies of other hantaviruses, such as Black Creek Canal, Andes, Puumala, Seoul, and Hantaan viruses have suggested that hantavirus transmission likely occurs through direct contact between infected and naïve animals rather than through contact with contaminated excreta and/or secreta, which is thought to be the primary means of transmission to humans [14,15,16,17,18,19].

SNV infections have classically been performed via intramuscular (IM) infection, as this route is thought to most closely mimic the natural infection of deer mice through biting [12,20]. Additionally, because SNV is thought to be transmitted primarily by male mice, these mice have been used exclusively for virus propagation. The ability of SNV to replicate to high titers following other routes of infection or in the tissues of female and/or deer mice of varying ages has not been determined. The propagation of viruses within specific rodent hosts rather than in cell cultures requires a consistent and reliable protocol to generate a high-titer virus while using as few animals as possible. Therefore, we sought to determine whether IM infection results in higher levels of SNV than intraperitoneal (IP) infection in experimentally infected deer mice. We also sought to identify age- and/or sex-related differences that may have a significant effect on viral replication and subsequent SNV production. These initial experiments were performed because of their implications in subsequent studies involving the SNV infection of deer mice.

We further sought to determine whether the main mechanism of SNV transmission between deer mice is via direct interaction between mice or through exposure to contaminated excreta/secreta. Because of the temporal nature of SNV shedding and incidence of infection, we set out to determine what mechanisms might contribute to increased SNV replication and shedding in infected deer mice leading to increased transmission rates. To this end, we tested our hypothesis that the induction of a heat shock response in SNV-infected deer mice or increases in testosterone levels of SNV-infected male deer mice will lead to increases in virus replication, shedding, and transmission.

## 2. Materials and Methods

### 2.1. Ethics Statement

The experiments described were carried out at the National Microbiology Laboratory (NML) of the Public Health Agency of Canada. All the animal experiments were approved by the animal care committee at the Canadian Science Center for Human and Animal Health in accordance with the guidelines provided by the Canadian Council on Animal Care. *Peromyscus maniculatus rufinus* (deer mice) used for all the experiments were provided by a breeding colony housed at the University of Manitoba. The University of Manitoba breeding colony was established with deer mice brought in from a previously established breeding colony at Rocky Mountain Laboratories in Montana, USA, which had been established from deer mice obtained from a breeding colony at the University of New Mexico [21]. All the deer mice from the colony were seronegative and Sin Nombre virus free.

### 2.2. Animals and Infections

The deer mice were provided by a breeding colony housed at the University of Manitoba. All the incoming animals were acclimated for at least one week before the experimental procedures began. All the animal work and infections were performed under BSL-4 conditions at the NML. The animals were given food and water ad libitum and monitored daily throughout the course of the experiments. The deer mice were infected intramuscularly with the equivalent of 2 × 10^5^ genome copies of SNV (77734). The SNV strain 77734 is the original genotypically matched SNV strain for the subspecies of deer mice used by our group. The virus was originally isolated from a single wild *P. maniculatus rufinus* and used for the inoculation of deer mice in the original description of the experimental SNV infection of this species [20]. The virus was passaged only in vivo within deer mice.

### 2.3. Viral Stock Preparation

The lungs from the infected deer mice were harvested at 10 days post-infection, homogenized via manual tissue homogenizers (VWR catalogue #47732-450), and clarified by low-speed centrifugation twice for 15 min at 525× *g*. Final lung homogenates were prepared in 1 mL of Dulbecco’s modified eagle medium (DMEM) per set of lungs, divided into 500 µL aliquots, and placed at −80 °C for future infections. A total of 140 µL was taken from each homogenate for RNA extraction and viral RNA copy number determination by RT-qPCR.

### 2.4. Transmission Experiments

For direct, intra-cage transmission, 1 infected deer mouse was housed with 2–4 naïve, uninfected sex-matched deer mice for 6 weeks. Subsequently, 2–3 weeks following infection, infected seeder mice were bled retro-orbitally using heparinized capillary tubes to confirm SNV infection by qRT-PCR. Then, 6 weeks post-infection, all the mice were euthanized, and blood and lung samples were taken to determine the presence of SNV and/or seroconversion. To determine if heat shock responses increase the SNV transmission rate, identical experiments were carried out with the exception that each SNV-infected deer mouse was given paeoniflorin (50 mg/kg) via oral gavage every 2 days for 3 weeks following infection. To determine if the level of testosterone affects the transmission rate, identical experiments were carried out with the exception that each SNV-infected deer mouse was castrated and implanted with an osmotic pump delivering either testosterone enanthate (200 µg/day for 28 days) or vehicle.

For indirect transmission, individual deer mice were infected with SNV and housed independently for 3 weeks. Dirty deer mouse cages were not changed during the second and third week of SNV infection. At 3 weeks post-infection, infected deer mice were removed from their cages and 3 naïve, uninfected deer mice were placed in each cage for 1 week before being separated to prevent any intra-cage transmission. Then, 3 weeks post-exposure to the dirty cages, all the animals were euthanized, and blood and lung samples were taken to determine the presence of SNV and/or seroconversion.

### 2.5. Blood, Swab, and Tissue Collection

All the mice were exsanguinated via cardiac puncture under isoflurane anesthesia before being euthanized. Either whole blood in K2-EDTA tubes or serum was collected. Oral and rectal swabs were taken using fine tip rayon swabs (MWE cat #MW113) and placed in 500 µL of DMEM. Pieces of lung, heart, and spleen were collected in 1 mL of RNAlater for the detection of SNV RNA. For urine collection, deer mice were housed individually in sterile cages with wire bottoms overnight. Any feces and urine were collected from the bottom of the cage the following morning.

### 2.6. Detection of Viral RNA

After 24 h in RNAlater, the collected tissues were removed from RNAlater, homogenized in 600 µL RLT lysis buffer (Qiagen, Hilden, Germany), clarified by centrifugation, and diluted to 30 mg equivalents in RLT lysis buffer. RNA was extracted using an RNeasy mini kit (Qiagen). RNA from whole blood, swabs, and urine were extracted using a Viral RNA mini kit (Qiagen). RT-qPCR detection of SNV S segment was performed on a QuantStudio 3 instrument (Applied Biosystems, Foster City, CA, USA) using a one-step protocol using a Quantitect probe RT-PCR kit (Qiagen) per the manufacturer’s instructions in triplicate (SNVforw—GCAGACGGGCAGCTGTG; SNVrev—AGATCAGCCAGTTCCCGCT; SNVProbe—5’FAM-TGCATTGGAGACCAAACTCGGAGAACTC-3’IAbkFQ). RT-PCR was carried out in 3 stages: reverse transcription (50 °C for 30 min), Taq activation (95 °C for 15 min), and amplification (40 cycles of 94 °C for 15 s and 60 °C for 60 s). Data acquisition occurred at the end of the annealing/extension stage (60 °C for 60 s) of each amplification cycle. A standard curve ranging from 5 × 10^7^ to 5 copies of in vitro transcribed SNV S segment RNA was used to calculate the copy number per mL or mg of tissue for each sample by interpolation. A Ct cut-off value of 35 was used for determining positive samples, as this Ct value corresponded to a copy number of less than 1.

### 2.7. Determination of Seroconversion by ELISA

An ELISA was performed to confirm or assess the seroconversion of the infected and exposed deer mice. Antibodies specific for SNV cross-react with antigens from Black Creek Canal (BCC) virus (Hjelle, 1997). Therefore, 96-well, half-area, high-binding polystyrene plates (Corning, Corning, NY, USA) were coated with BCC virus lysate at 50 ng per well and incubated overnight at 4 °C. The following day, the plates were washed with PBS-T and then blocked with 5% skim milk in PBS-T (PBS + 0.1% Tween 20) for 1 h at 37 °C. The serum samples were diluted 1:100 in 5% skim milk in PBS and added to PBS-T washed plates in triplicate overnight at 4 °C. The following day the plates were washed with PBS-T and secondary HRP-conjugated anti-Peromyscus leucopus antibody (KPL; 1:1000) was added to the plates for 1 h at 37 °C. The plates were washed with PBS-T, and ABTS substrate (Thermofisher, Waltham, MA, USA) was added and incubated for 30 min before reading the OD values at 405 nm. Positive samples were those that had an OD greater than the mean OD plus 3 standard deviations seen in the negative control wells.

### 2.8. Induction of Heat Shock Responses

The deer mice were given either 500 mg/kg geranylgeranylacetone (GGA) or 50 mg/kg paeoniflorin (PFL) in 100 µL via oral gavage using plastic feeding needles. Then, 8, 24, and 48 h following treatment, the deer mice were euthanized, and blood and tissues were collected to determine the expression levels of heat shock protein 70 (HSP70).

For assessment of long-term HSP70 mRNA levels, groups of deer mice were given 50 mg/kg PFL via oral gavage daily for 1 week. Groups of deer mice were euthanized following 8 h and at 4 days, 8 days, and 12 days following the onset of treatment for tissue collection.

### 2.9. Detection of Heat Shock Protein mRNA Expression

Tissues were collected in RNAlater. For the determination of the HSP70 mRNA levels, the tissues were removed from RNAlater, and RNA was extracted from the tissues using an RNeasy plus mini kit (Qiagen) following the manufacturer’s instructions. Then, 100 ng of extracted tissue RNA was used in a two-step qRT-PCR reaction in triplicate performed on a StepOne Plus instrument (Applied Biosystems). For reverse transcription, a superscript III RT first strand synthesis kit (Invitrogen, Carlsbad, CA, USA) was used, followed by qPCR using PowerUp SYBR Green Master Mix (Applied Biosystems). For reverse transcription, 100 ng of template RNA was mixed with random hexamers as primers in a 20 µL reaction for 5 min at 65 °C followed by 10 min at 25 °C and 50 min at 50 °C. Then, 2 µL of cDNA was used for qPCR. The cycle parameters for qPCR were 2 min at 50 °C and 2 min at 95 °C followed by 40 cycles of 3 s at 95 °C and 30 s at 60 °C. The qPCR reactions were 20 µL with all the oligonucleotide concentrations at 2 µM. The fold change in gene expression of HSP70 was calculated using the ΔΔCt method with *GAPDH* as a reference gene. Primers for HSP70 (F- GCGGGTGGCGTGATGA; R- GAAGATCTGCGTCTGCTTGGT) and GAPDH (F- TCCGTCGTGGATCTGACATG; R- ACGCCTGCTTCACCACCTT) were also obtained.

### 2.10. Castration of Male Deer Mice

To eliminate most endogenous testosterone, male deer mice were castrated according to a procedure modified from Valkenburg et al [22]. The anesthetized mice were shaved and cleaned with chlorhexidine and 70% ethanol. The mice were then maintained under isoflurane anesthetization and secured to a heating pad for the duration of the surgical procedure. The mice were also given meloxicam (2 mg/kg subcutaneous) before the start of the procedure. With a sterile scalpel, a small (less than 1 cm) incision was made to the skin and peritoneum adjacent to the rectum on both sides. Using sterile forceps, the peritoneum was opened, and the testicular fat pad was pulled out of the peritoneal opening with a second pair of sterile forceps. Using hemostats, the fat pad, testes, and epididymis were clamped off and the testes removed. The remaining vasculature was then allowed to clot and placed back into the opening in the peritoneum and skin. The incisions were then glued shut using veterinary glue. The same procedure was then repeated on the other testes, and the deer mice were permitted to recover in their own cages until fully alert. The animals were monitored daily for any signs of stress or opening of the surgical incisions. The animals were also administered meloxicam (2 mg/kg subcutaneous) daily for 3 days following the surgical procedure.

### 2.11. Testosterone ELISA

Levels of testosterone were determined using a testosterone ELISA kit (Enzo Life Sciences, Farmingdale, NY, USA) according to the manufacturer’s instructions. Briefly, deer mouse serum was collected and diluted 1:40 in sample assay buffer and incubated with an anti-testosterone antibody for 1 h at room temperature in 96-well assay plates. The wells were then incubated with an alkaline phosphatase–testosterone conjugate for 1 h at room temperature before washing and adding a pNpp substrate for an additional hour. A stop solution was added, and absorbance readings were taken at 405 nm. The absorbance levels were inversely proportional to the testosterone concentration in each well. The serum testosterone levels were determined using a standard curve of known testosterone concentration.

### 2.12. Implantation of Osmotic Pumps

For the administration of exogenous testosterone to the castrated male deer mice, osmotic pumps (model 1004, Alzet) were filled with either propylene glycol or testosterone enanthate and implanted into the deer mice subcutaneously as per the manufacturer’s instructions. Briefly, the castrated male deer mice were anesthetized and shaved. A small incision in the right flank of the body of each mouse was made, and pumps were implanted between the skin and peritoneum. The incisions were then held together with surgical staples that were removed 7–8 days following pump implantation. The animals were administered meloxicam (2 mg/kg subcutaneous) following pump implantation.

### 2.13. Statistical Analysis

All the results were analyzed and graphed using Prism 5 software (Graphpad, La Jolla, CA, USA). The statistical significance between the groups was determined using a Mann–Whitney test, one-way analysis of variance (ANOVA), or Fisher’s exact test, where appropriate. *P* values have been included in the relevant figures for comparisons that resulted in statistically significant differences.

## 3. Results

### 3.1. Refinement of SNV Infection in Deer Mice

**Figure 1:**
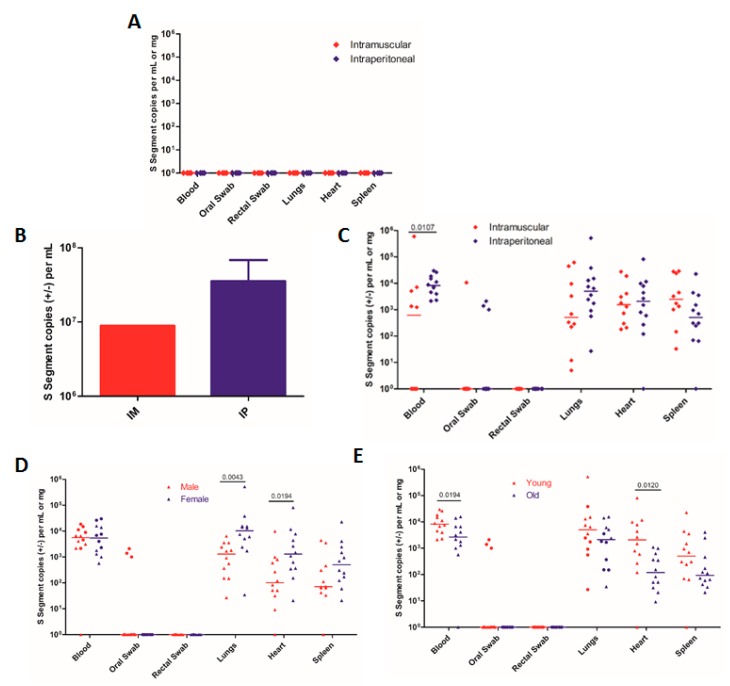
Sin Nombre virus (SNV) replication and shedding in male and female deer mice of different ages. (**A**) Groups of 1–2-month-old deer mice were inoculated with a VeroE6-adapted SNV. *n* = 4. (**B**) and (**C**) Groups of 10 (intramuscularly (IM) infected) or 12 (intraperitoneally (IP) infected) deer mice were infected with SNV, and S segment copies were detected in (**B**) pooled lung homogenates from each group for producing viral stocks or (**C**) each individual mouse to determine the amounts of viral replication and shedding. (**B**) 1 IM group was tested, and 3 IP groups were tested. (**D**) and (**E**) Comparison of replication and shedding seen in (**D**) male vs. female deer mice 10 days post-infection (circles: 1–2-month-old deer mice; triangles: 5–6-month-old deer mice) or (**E**) 1–2-month-old (young) vs. 5–6-month-old (old) deer mice 10 days post-infection (circles: male deer mice; triangles: female deer mice). The data shown are the mean + SEM (**B**) and medians (**C**–**E**) for each group. The numbers indicate the p value as assessed by a Mann–Whitney test.

We first confirmed that a Vero E6-adapted SNV (77734, herein referred to as VE6-SNV) is unable to cause productive infection in deer mice, underscoring the need for working SNV stocks produced in natural rodent hosts (Figure 1A). A Vero E6-adapted SNV was given to deer mice either via IM or IP infection. SNV RNA was undetectable in any of the tissues or swabs tested 10 days post-infection (Figure 1A). Following this confirmation, a deer mouse-only-passaged SNV was used. To determine whether IM infection results in higher levels of SNV replication than IP infection, groups of deer mice (IM: *n* = 10, IP: *n* = 12) were infected with SNV (strain 77734). The animals were euthanized on day 10, during the acute phase of infection, which was previously determined to be an optimal time for tissue collection and virus preparation due to peak viral loads [13,20]. Pooled lung homogenate from IM infected mice produced a viral stock with an RNA copy number that trended lower than those seen from three separate pooled groups of IP infected mice, but this did not reach statistical significance due to the production of only one stock from the IM infected animals (Figure 1B). This may indicate that the IP infection route results in higher SNV levels when using pooled lung homogenates for virus preparation, but further experiments should be performed. IM infected deer mice were also less likely to have detectable viral RNA in their blood compared with IP infected mice, with only 50% of IM infected mice having detectable SNV RNA in their blood versus 100% of IP infected mice at day 10 (Figure 1C). Although not statistically significant, IM infected mice seemed to have lower levels of SNV in the lungs (Figure 1C). Overall, it appears that IP infections produce equal or greater levels of SNV than IM infections and provide more consistent viremia.

We then sought to determine whether the age or sex of the infected deer mice affects SNV replication. Groups of juvenile (1–2 month) and adult (5–6 month) deer mice of either sex (*n* = 6) were infected via IP infection. We did not see any difference in levels of SNV RNA in the blood between male and female mice (Figure 1D), but interestingly, we found significantly higher levels of SNV RNA in the lungs and hearts but not the spleens of female mice (Figure 1D). The significant difference in SNV RNA in these tissues seemed to be due to differences in viral replication between sexes in younger mice. Controlling for age, younger females had higher levels of SNV in both the lungs and heart (*p* = 0.0194 and *p* = 0.0260, respectively, Mann–Whitney test; Figure 1D). We also found that younger deer mice had higher levels of SNV RNA in the blood than older deer mice (Figure 1E), as well as higher levels of viral RNA in the heart (*p* = 0.0120, Mann–Whitney test; Figure 1E). However, when controlling for differences in sex, we saw no significant differences in viral loads in the blood or tissues assayed (Mann–Whitney test, Figure 1E). In terms of viral shedding, we were only able to detect SNV RNA in the oral swabs of three male mice, while no rectal swabs produced detectable levels of viral RNA (Figure 1E). Overall, the data suggest that there may be small differences in viral replication between younger and older or male and female deer mice, but these differences will likely have a negligible effect on the overall amount of SNV produced when preparing virus stocks from infected animals.

### 3.2. Direct Intra-Cage Transmission of SNV

We first wanted to determine how readily SNV transmits between deer mice following direct contact between an infected deer mouse and its uninfected cage mates and to determine whether indirect transmission via contact with contaminated excreta can occur. Previous studies examining the transmission of SNV showed either a very low transmission rate of 1/54 exposed animals becoming infected [11] or did not examine the primary mechanism(s) of transmission that did occur [12]. Here, 33 naïve deer mice across 11 cages were exposed to a sex-matched, SNV-infected deer mouse for 6 weeks. Following 6 weeks of SNV exposure, 8 uninfected deer mice (24%) had either seroconverted or were qRT-PCR positive for SNV RNA (Table 1), which is a transmission rate similar to the highest rate seen in the experiments done by Bagamian et al. [12]. Here, we demonstrated that a model that can consistently produce a high rate of experimental SNV transmission in a controlled laboratory setting. It is also possible that there was an underestimation here of the number of transmission events in that the deer mice that were exposed to SNV may have been euthanized before seroconverting or developing a productive infection.

We next wanted to determine whether uninfected deer mice could contract SNV following exposure to bedding and caging that had been contaminated with SNV while housing SNV-infected deer mice. Historically, using our IM SNV infection model, we see peak viral loads at around 14 days post-infection (dpi) with viral copy numbers in the blood and tissues gradually dropping through 28 dpi. Therefore, we used this as a guideline, and we housed the infected deer mice for 21 days and did not change the soiled cages between 7–21 dpi. On day 21, the SNV-infected deer mice were removed from their cages, and 3 uninfected deer mice were placed in each soiled cage for 1 week before being separated to prevent any intracage transmission. Following housing in the contaminated cages and exposure to the contaminated bedding, none of the 30 deer mice across the 10 cages became infected with SNV (Table 1). Therefore, there is a significantly increased risk of deer mice becoming infected while being directly exposed to an infected cage mate than from potentially SNV-contaminated bedding (relative risk = 1.320 (1.088–1.601), *p* = 0.0051 (Fisher’s exact test)). While it is difficult to rule out the possibility of transmission occurring through exposure to contaminated excreta and/or secreta in this type of indirect manner, our data strongly suggests that the transmission of SNV between deer mice occurs via direct contact between infected and uninfected individuals.

### 3.3. Induction of Heat Shock Responses

When infected with SNV, deer mice become persistently infected, typically throughout the lifetime of the individual. During long-term infection, SNV is able to persist and replicate at low levels in a variety of tissues including the lungs, heart, brown adipose tissue, liver, kidney, and spleen among others [13]. Throughout the lifetime of infected deer mice, it is thought that there may be periodic episodes of recrudescence during which SNV replicates to higher levels [13]. It has been suggested that cold stress is able to induce SNV replication in vivo and that this is accomplished through the expression of heat shock family proteins that are responsible for the transportation of the uncoupling protein from the cytosol to the mitochondria [23,24]. Heat shock proteins (HSPs) have also been shown to aid in the replication of several other families of viruses, and there is evidence that HSP70 is able to interact with the nucleocapsid protein of HTNV [25,26]. Therefore, we wanted to determine whether the induction of heat shock responses and the expression of heat shock family proteins in SNV-infected deer mice can lead to increased levels of viral replication and shedding, leading to higher rates of SNV transmission.

**Figure 2:**
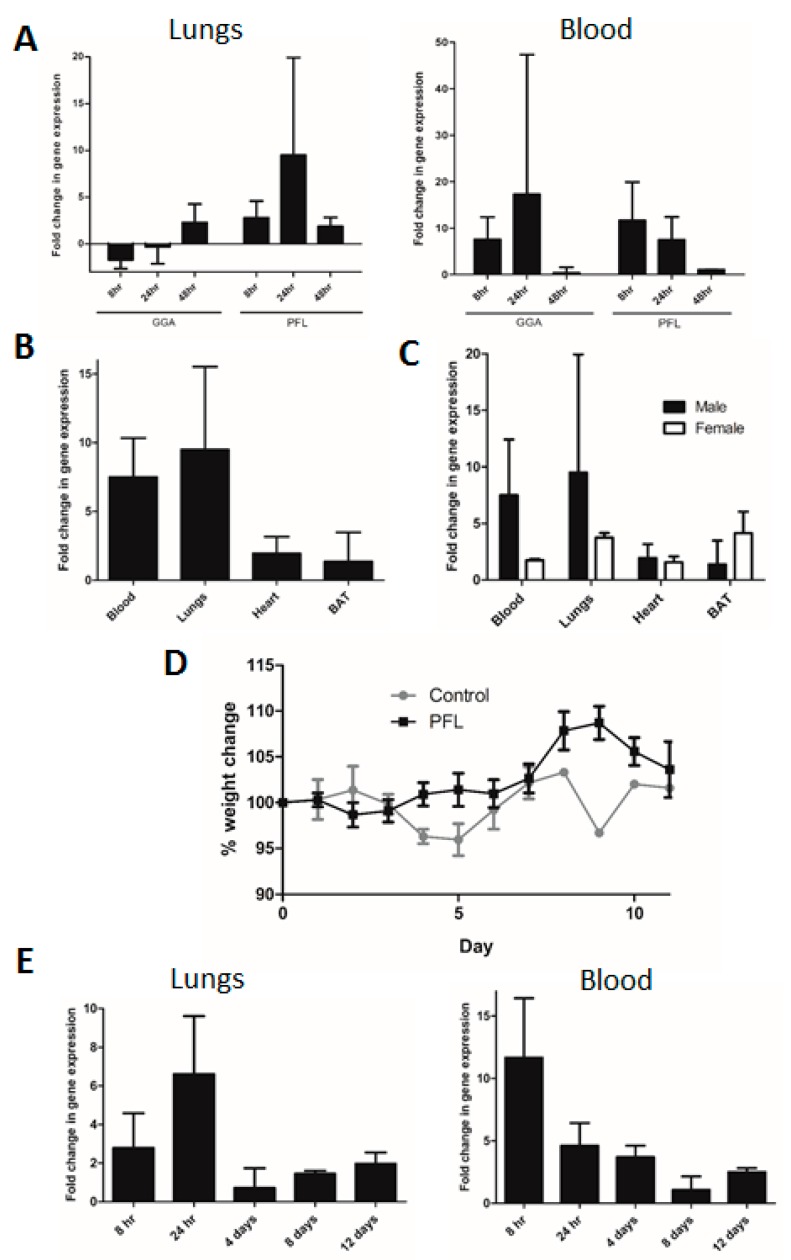
Induction of heat shock protein 70 (HSP70) expression in deer mice. (**A**) Levels of HSP70 expression were assessed in either the blood or the lungs as compared with the vehicle-treated mice. *n* = 3. (**B**) Mice were given PFL, and HSP70 expression was assessed 24 h later in tissues where SNV persistence has been shown to occur. *n* = 3. (**C**) HSP70 expression in various tissues in both male and female mice 24 h following oral gavage of PFL. *n* = 6. (**D**) Change in weight following daily doses of PFL for 7 days. *n* = 12. (**E**) HSP70 expression levels at different time points in the blood and lungs of deer mice following daily PFL administration. *n* = 12.

We tested two compounds, geranylgeranylacetone (GGA) and paeoniflorin (PFL), which have been shown to induce heat shock responses, to determine whether they increased the HSP70 mRNA transcription in deer mice [27,28,29,30,31]. Mice were given either GGA or PFL, and the fold change in the mRNA expression compared with that of the vehicle-treated animals was reported. The treated deer mice had increased HSP70 mRNA levels 24 h following the administration of each drug (Figure 2A). PFL-induced HSP70 mRNA increases in the blood, lungs, heart, and brown adipose tissue of the treated mice, tissues known to be reservoirs for SNV during persistent infection [13] (Figure 2B). There was no significant difference in the HSP70 levels in treated male or female deer mice (two-way ANOVA; Figure 2C). To determine if continuous administration of PFL had any adverse effects, treated deer mice were monitored and weighed daily for up to 12 days while being administered PFL on a daily basis for 7 days. No adverse signs or weight loss were seen in the treated or control deer mice (Figure 2D). Treatment of the deer mice with PFL daily seemed to promote a tolerant effect, as the levels of HSP70 mRNA peaked between days 1 and 4 and fell to control levels between days 4 and 7 of administration (Figure 2E). These results suggest that the administration of PFL can induce an increase in HSP70 mRNA levels and a heat shock response in treated deer mice.

**Figure 3:**
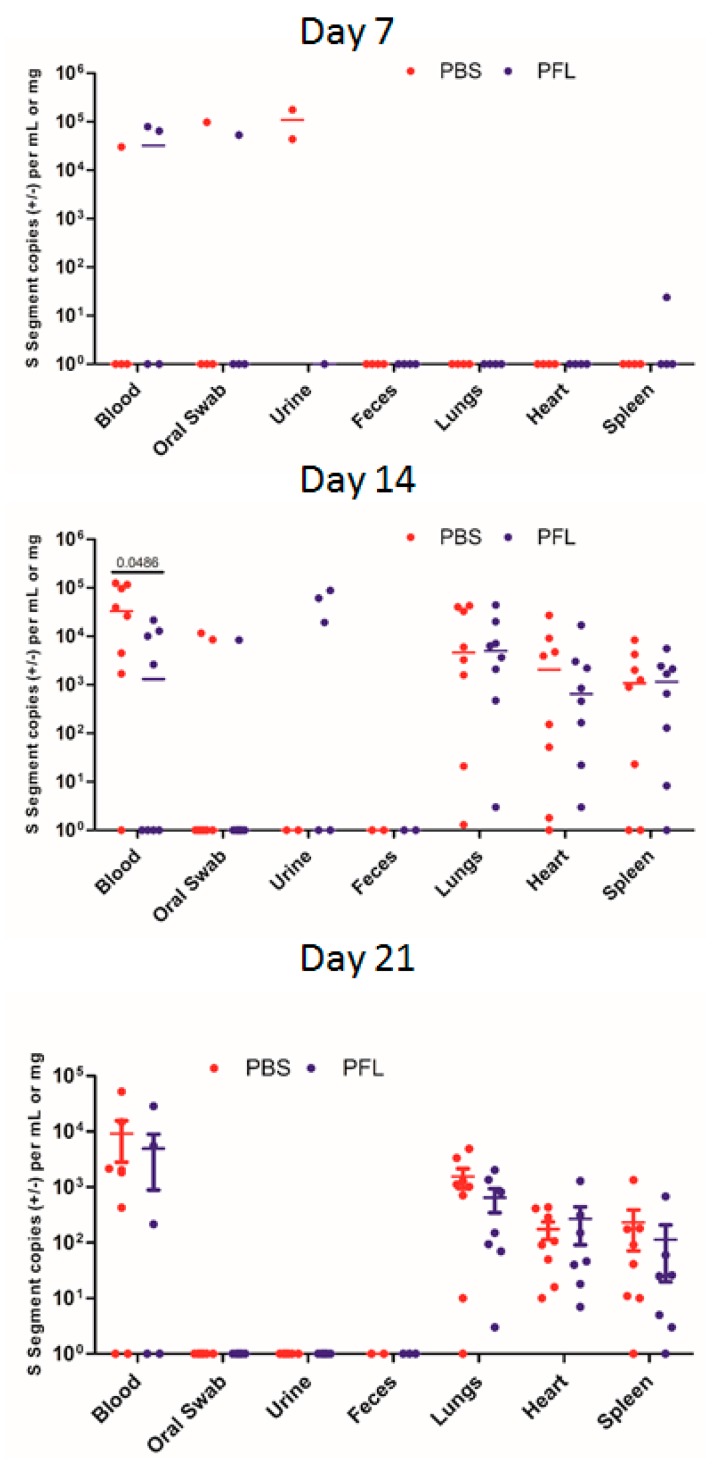
Replication and shedding of SNV in control and paeoniflorin (PFL)-treated deer mice. The SNV viral copy number was assessed in the tissues and samples listed at the indicated time points post-infection. The numbers indicate the p value as assessed by a Mann–Whitney test.

Next, we wanted to determine whether treatment with PFL during SNV infection is able to increase replication of SNV leading to higher levels of viral shedding and rate of SNV transmission. Due to the tolerance effect seen in our preliminary studies, PFL or PBS was given to SNV-infected deer mice once every 2 days for 14 days. Oral gavage with PFL did not increase the amount of viral RNA copies seen in the blood or tissues of the infected deer mice (Figure 3). During the peak of acute infection, day 14, there was no significant difference in the viral genome copy number in the lung, heart, or spleen, and only slightly higher copy numbers in the blood of the PBS-treated control deer mice compared with the blood of the PFL-treated mice were detected (Figure 3). Interestingly, we were able to detect SNV RNA in the urine of three PFL-treated deer mice at day 14, while we were not able to do so in any of the control animals; however, SNV RNA was detected in urine at day 7 in two of the control animals (Figure 3). Considering the data, a further exploration into the effects of PFL on viral shedding in excreta and urine should be considered.

Concurrently, we wanted to determine whether the treatment of SNV-infected deer mice with PFL had any effect on the rate of SNV transmission. The infected deer mice were housed with uninfected deer mice for 6 weeks. Because we did not see a rise in the viral RNA copy numbers until the second week of infection, each infected deer mouse was treated every 2 days with PFL for 21 days to induce the expression of heat shock proteins. Following 6 weeks of exposure to the infected deer mice, 16 out of 37 deer mice (43%) were either seropositive or qRT-PCR positive (Table 2). The treatment of the infected deer mice with PFL led to an increase in the percentage of naïve deer mice becoming infected, although this did not reach statistical significance as compared with the untreated animals (Table 2; relative risk = 1.355 (0.9489–1.878), *p* = 0.1311(Fisher’s exact test)). While it remains to be seen whether other means of heat shock induction in SNV-infected deer mice might increase SNV replication and transmission, our treatment did not have a significant effect on the course of acute SNV infection and the ability to transmit SNV in deer mice.

### 3.4. Transmission Rates in Male and Female Deer Mice

It is thought that males differentially contribute to SNV transmission due to their wider habitat range and higher seropositivity in field studies [32,33]. We wanted to look at whether there is any intrinsic difference between male and female deer mice when it comes to SNV infection and transmission. Across the experiments described above, there were a total of 31 uninfected exposed male deer mice in contact with an infected male deer mouse and 39 uninfected, exposed female deer mice in contact with an infected female. 11 male and 13 female deer mice became infected (35% and 33%, respectively), and there were no significant differences seen in the rates of transmission between males and female in the untreated and PFL-treated groups (Table 3). There was an increase in the number of males that became infected in the PFL-treated group as compared with the untreated group (15% compared with 50%), and this resulted in a slight increase in the risk of infection for the uninfected animals (relative risk = 1.692 (1.009–2.838), *p* = 0.0656 (Fisher’s exact test)). Therefore, while transmission in natural settings may be carried out predominantly by infected male deer mice, there is no intrinsic, sex specific difference in the course of SNV infection and in the ability of male and female deer mice to transmit SNV.

### 3.5. Effect of Testosterone on SNV Infection

**Figure 4:**
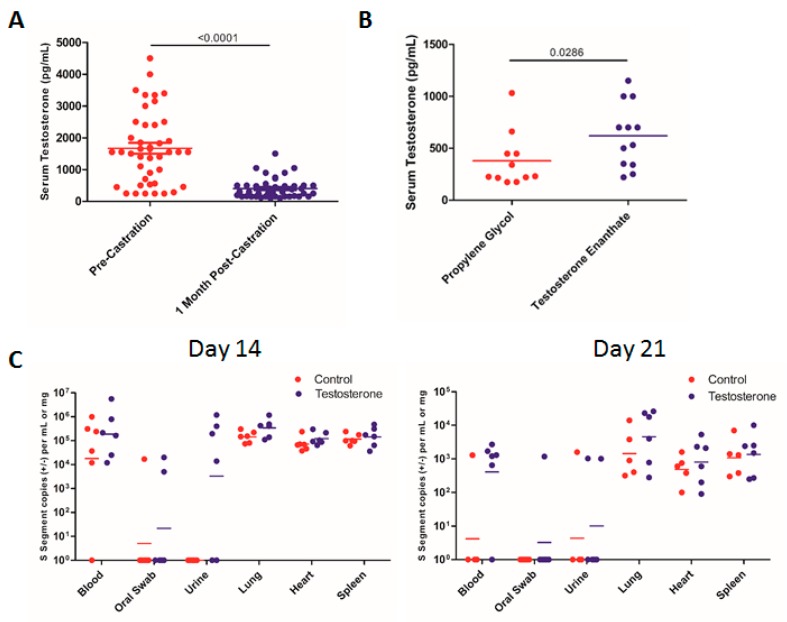
Replication and shedding of SNV in castrated male deer mice given testosterone. (**A**) Serum testosterone levels in deer mice before and 1 month following castration. (**B**) Serum testosterone levels in deer mice implanted with osmotic pumps containing either testosterone enanthate or the control. (**C**) The SNV viral copy number was assessed in the tissues and samples listed at the indicated time points post-infection. The numbers indicate the p value as assessed by a Mann–Whitney test.

In addition to field evidence that shows that male rodents are more commonly infected with hantaviruses than females, there is evidence that wounding and sex hormone levels may play a role in hantavirus replication, persistence, and transmission [34]. Elevated testosterone levels have been linked to increased wounding and rates of Seoul (SEOV) infection in male Norway rats [35]. Additionally, experimental gonadectomy results in an increase in SEOV titers in female rats but a decrease in male rats, and this is dependent upon the modulation of the innate immune system under the control of sex hormones [36]. Corticosteroids can also influence hantavirus infection through the alteration of the immune response of the host [37]. There is substantial evidence that sex hormones play a significant role in immune system function, and several studies on the role of the immune system in mediating hantavirus persistence in host species have been performed [38,39]. Because males are generally thought to contribute to the majority of SNV transmission, we sought to determine the role of testosterone in influencing SNV infection in male deer mice. We castrated deer mice and confirmed that testosterone levels in these mice were sufficiently depleted 1 month after the castration (Figure 4A). Then, 1 month following the castration, the deer mice were implanted with osmotic pumps, which delivered either propylene glycol or testosterone enanthate (200 µg/day) for 28 days, and infected with SNV. At 14 and 27 dpi, the SNV copy numbers did not differ significantly between the deer mice receiving testosterone or the control (Figure 4C). We were able to detect SNV RNA in the urine and blood more frequently in the testosterone group compared with the controls, but this difference did not reach statistical significance (Fisher’s exact test; Figure 4C). Among the deer mice implanted with osmotic pumps, the serum testosterone levels were variable, yet significantly higher than in the control animals (Figure 4B). Issues with the efficiency of the osmotic pumps may have resulted in lower serum testosterone levels in some animals; however, SNV RNA levels did not correlate with serum testosterone levels at either time point.

To determine whether the presence of the testosterone had any impact on the transmission of SNV, castrated deer mice were again implanted with osmotic pumps containing testosterone or propylene glycol and housed with uninfected mice for 6 weeks. Here, we saw no difference in the rate of transmission between the groups of deer mice housed with the infected animals receiving testosterone or the control (Table 4; *p* = 0.7892 (Fisher’s exact test)). Interestingly, we found no significant difference between the groups containing seeder mice receiving testosterone or the control and our initial, direct, intracage transmission experiments as well. These data indicate that the depletion of testosterone via castration or the presence of testosterone does not influence SNV replication, shedding, or the transmission rate.

## 4. Discussion

The worldwide occurrence of hantavirus infections in humans is due to the widespread nature and evolution of each virus’s respective host. In general, each virus is restricted to its host species and does not cause overt disease in that host. There are certain characteristics of hantaviruses, which make them difficult to study experimentally. The nature of the evolutionary relationship between each virus and its host means that each particular host species is needed for laboratory-based studies. The need for BSL-3 and BSL-4 facilities to undergo animal studies on these viruses makes studying various ecological aspects challenging as well. Additionally, the fact that certain hantaviruses acquire mutations following in vitro culture that eliminate their capacity to infect not only their specific rodent host, but also other animals used for pathogenesis studies makes working with these viruses and propagating them difficult [8,9,10]. These issues have made understanding some ecological aspects of SNV challenging. There have, therefore, been a limited number of studies on the transmission of SNV among deer mice. Previously, it was thought that direct contact between infected and uninfected deer mice rarely led to the transmission of the virus in experimental settings and field data could only provide indirect evidence of the mechanisms of transmission. Attempts to define these mechanisms have not seen much success, and only a single study has yielded a significant number of transmission events [11,12].

To address the issue of needing a more reliable method of producing viral stocks in vivo, our goal was to refine our SNV infection model to maximize the amount of SNV collected for viral stocks from tissues of infected deer mice. We were able to show that IP infection produces viral stocks of roughly equivalent RNA copy numbers from pooled lung homogenates and appears to be a more reliable infection route compared with IM infection. We found subtle differences in the levels of SNV RNA in the blood and tissues between certain groups of mice, but these differences could be accounted for when controlling for the sex or age of the mice. Overall, similar levels of SNV replication and production are seen regardless of the age and sex of the infected deer mice. Here, we provide evidence that viral stocks of high RNA copy numbers can be achieved through the infection of either sex at a variety of ages. This has implications not only for our SNV infection model and virus preparation, but also for other hantaviruses that need to be passaged in their natural rodent host to maintain virulence. In addition, by experimentally showing that the SNV infection of deer mice of both sexes at different stages of life does not result in significantly different infection kinetics, we were able to expand the range of animals available for use in future infection and transmission studies of SNV in deer mice.

As noted above, the transmission of SNV has not been well characterized, with attempts to study it providing either no assessment of the mechanism(s) of transmission [12] or yielding very low transmission rates [11]. Very few experimental studies of SNV transmission have been conducted. Our goal was to determine whether SNV does, in fact, transmit directly between deer mice or whether indirect transmission can occur via exposure to SNV found within contaminated caging. We were able to detect SNV transmission in 24% of exposed, uninfected deer mice that had been housed with a sex-matched, infected cage mate for 6 weeks. This is in line with the percentage of exposed deer mice that became infected in experimental outdoor enclosures over a similar timeframe [12]. We then exposed uninfected deer mice to cages that had housed SNV-infected deer mice for 3 weeks following SNV infection. To allow for the best chance of SNV shedding leading to transmission, the cages were not changed after 7 days post-infection. Following exposure to contaminated cages for 1 week, no uninfected deer mice contracted SNV. This is in contrast to the data shown for other hantaviruses, whereby the virus is secreted readily in urine, feces, and saliva and can contribute to transmission [14,15,16,17,18,19]. Interestingly, this is also the main mechanism that is thought to contribute to the transmission of SNV to humans. The acute phase of SNV infection in this experiment is likely to lead to the highest levels of SNV shedding, so it is unlikely that this mode of transmission plays a major role in the natural transmission of SNV. This is in contrast to Puumala virus (PUUV), an HFRS-causing virus that has been well studied, relatively speaking, compared with SNV. PUUV has been shown to transmit readily between bank voles, the virus’s natural reservoir, and multiple studies have examined and reviewed the virus’s ability to be shed throughout the lifetime of bank voles, as well as to persist in the environment and transmit to naïve animals [17,18,40,41,42]. We feel as though the data seen here fits with the relatively rare seropositivity seen in wild deer mice, as well as the epidemiological data regarding human SNV cases in North America. While we cannot definitively state that indirect transmission of SNV between deer mice through contaminated excreta/secreta does not occur, it is likely that direct contact between deer mice is the major driver of SNV transmission. Due to the difficulty of determining when the highest levels of live SNV are shed during infection, it is difficult to establish when the best time for exposure to contaminated bedding should occur. There is also evidence that infection kinetics and the shedding of hantaviruses in wild animals differs from what is seen during experimental infection, which may play a role in the outcome of these types of studies. Experiments employing co-housed deer mice separated by some physical barrier that prevents direct contact but allows for viral particle transmission could provide interesting results in this regard.

In our infection model, peak SNV replication occurred around 14 days post-infection and then began to wane, resulting in variable levels and less reliable detection of SNV in the blood and tissues of infected deer mice more than 1 month following infection. This acute stage of infection likely provides the greatest chance of detecting high rates of transmission in deer mouse populations; however, the persistent, lifelong nature of SNV infections and periodic episodes of viral replication and shedding suggest that there are occurrences throughout the lifetime of infected deer mice that may lead to periods of increased transmission risk [13]. We hypothesized that heat shock responses and increased sex hormone levels may be two potential occurrences that will lead to greater levels of SNV replication, shedding, and higher transmission rates. Although we were able to induce heat shock responses assessed via relative levels of HSP70 mRNA, the administration of PFL every other day during acute SNV infection did not have an effect on the viral copy number in the tissues or blood at any of the time points nor did it cause an increase in SNV shedding (Figure 3). The administration of PFL to infected deer mice did cause an increase in the number of uninfected deer mice that contracted SNV in our transmission model compared with the direct transmission seen in the untreated deer mice, but this did not reach statistical significance (Table 2). We also did not see any difference across all the experiments in the rate of SNV transmission between male and female deer mice (Table 3). Therefore, although males are predominantly infected and may contribute to SNV transmission more frequently than females due to factors such as wider habitat ranges and behavioral differences, there is no intrinsic difference in the ability of male or female deer mice to transmit SNV. This is in line with the data above showing that during acute infection of deer mice, SNV copy numbers do not differ between male and female animals.

We wanted to determine whether differences in testosterone levels would impact SNV infection and transmission among male deer mice [32,35,36]. The castration of male deer mice did indeed result in a depletion of endogenous testosterone 1 month after the surgical procedure (Figure 4). The supplementation of exogenous testosterone enanthate did not result in increased levels of SNV copy numbers in the blood or tissues of the infected deer mice (Figure 4). There was a higher incidence of SNV detection in the urine of the testosterone-supplemented animals, but this did not reach statistical significance (Figure 4). The supplementation of SNV-infected seeder mice during transmission experiments with testosterone also did not result in any difference in SNV transmission rate compared with cages containing control, testosterone-depleted animals as SNV-infected seeders (Table 4).

Deer mice that have been recently infected with SNV are more likely to shed the virus [43]. However, given the persistent long-term nature of SNV infection in deer mice whereby the virus is able to replicate at low levels and remain in tissues presumably for the lifetime of the individual, it is likely that some incidents result in an increase in viral replication that allows for the shedding and transmission of the virus. Environmental and host factors have been shown to play a role in the ecology of hantavirus infections, including SNV. Changes in climate, season, habitat, mast, and population size and density have all been hypothesized or shown to play a role in the frequency of hantavirus prevalence within its host population, transmission, and outbreak incidence [44,45,46,47,48,49]. We were unable to detect either changes in SNV replication or shedding or a higher rate of SNV transmission following heat shock induction in SNV-infected deer mice. As seasonal incidences of SNV are known to occur in deer mouse populations, we hypothesized that this induction might mimic seasonal changes and trigger non-shivering thermogenesis that may play a role in SNV infection [13]. Sex hormones in host species and their role in viral infections have been studied extensively, including during the Seoul virus infection of Norway rats [35,36]. In our acute infection model, the lack of or presence of testosterone did not have an effect on SNV replication or the transmission rate. During acute infection with SNV, which provides the highest levels of viral RNA in blood and tissues, as well as the greatest chance of transmission, we could not detect any difference in these instances. It may be possible that during long-term infection when SNV is at a low copy number, these interventions may lead to an increase in viral titers and transmission. Further investigations into SNV infection dynamics and transmission during persistent infection seem warranted. Nevertheless, here, we have provided the first reliable experimental transmission model for SNV in deer mice, which will be useful in future studies concerning SNV infection and transmission, including preventative measures and possible triggers leading to SNV outbreaks in deer mouse populations.

## Figures and Tables

**Table 1:** Direct and indirect transmission of SNV in deer mice.

Group	Exposed, Naïve Mice	Transmission Events *	% of Naïve Infected	Risk Ratio (95% CI)	*P* Value (Fisher’s Exact Test)
Direct Transmission	33	8	24	1.320 (1.088–1.601)	0.0051
Indirect Transmission	30	0	0		

* Seropositive or qRT-PCR positive.

**Table 2:** Transmission of SNV among untreated and heat shock-induced, infected deer mice.

Group	Exposed, Naïve Mice	Transmission Events *	% of Naïve Infected	Risk Ratio (95% CI)	P Value (Fisher’s Exact Test)
Direct Transmission	33	8	24	1.355 (0.9489–1.878)	0.1311
PFL Treated ^ψ^	37	16	43		

* Seropositive or qRT-PCR positive, ^Ψ^ Treated with PFL every 2 days for 3 weeks.

**Table 3:** Number of transmission events/exposed deer mice in male and female groups.

Group	Male	Female
Control	2/13	6/20
PFL-Treated	9/18	7/19
Total	11/31	13/39

**Table 4:** Transmission of SNV by deer mice with/without testosterone.

Group	Exposed, Naïve Mice	Transmission Events *	% of Naïve Infected	Risk Ratio (95% CI)	P Value (Fisher’s Exact Test)
Propylene Glycol	30	10	33	1.074 (0.7339–1.572)	0.7892
Testosterone	29	11	38		

* Seropositive or qRT-PCR positive.
